# A Low Complexity System Based on Multiple Weighted Decision Trees for Indoor Localization

**DOI:** 10.3390/s150614809

**Published:** 2015-06-23

**Authors:** David Sánchez-Rodríguez, Pablo Hernández-Morera, José Ma. Quinteiro, Itziar Alonso-González

**Affiliations:** 1. Institute for Technological Development and Innovation in Communications, Edificio Polivalente II, 2^a^ planta, Parque Científico y Tecnológico, Campus Universitario de Tafira, 35017 Las Palmas de Gran Canaria, Spain; E-Mail: itziar.alonso@ulpgc.es; 2. IUMA Information and Communications Systems, Edificio Polivalente I, Parque Científico y Tecnológico, Campus Universitario de Tafira, 35017 Las Palmas de Gran Canaria, Spain; E-Mails: pablo.hernandez@ulpgc.es (P.H.M.); josemaria.quinteiro@ulpgc.es (J.M.Q.); 3. Department of Telematic Engineering, University of Las Palmas de Gran Canaria, Campus Universitario de Tafira, 35017 Las Palmas de Gran Canaria, Spain

**Keywords:** WLAN indoor localization, weighted decision trees, received signal strength, orientation, sensor fusion

## Abstract

Indoor position estimation has become an attractive research topic due to growing interest in location-aware services. Nevertheless, satisfying solutions have not been found with the considerations of both accuracy and system complexity. From the perspective of lightweight mobile devices, they are extremely important characteristics, because both the processor power and energy availability are limited. Hence, an indoor localization system with high computational complexity can cause complete battery drain within a few hours. In our research, we use a data mining technique named boosting to develop a localization system based on multiple weighted decision trees to predict the device location, since it has high accuracy and low computational complexity. The localization system is built using a dataset from sensor fusion, which combines the strength of radio signals from different wireless local area network access points and device orientation information from a digital compass built-in mobile device, so that extra sensors are unnecessary. Experimental results indicate that the proposed system leads to substantial improvements on computational complexity over the widely-used traditional fingerprinting methods, and it has a better accuracy than they have.

## 1. Introduction

Location estimation has been a term of growing interest for years as lightweight mobile devices have become the standard in the real world. Many user applications for these devices need some notion of the current position, and hence, its development is one of the keys to the success of pervasive computing. Thus, location-aware services have made it possible to use applications capable of sensing their location and modifying their setting and functions accordingly [1]. A positioning system can be implemented in applications related to navigation, security, robotic industry, health and entertainment.

To date, many authors have worked on solving the problem of indoor location determination for both research and commercial interests. The research field has focused on different communication networks, such as WLAN, low-rate WPAN, infrared, ultrasound and, recently, visible light communications [2]. Hence, most research is based on WLANs, because they have been deployed with worldwide success.

Several types of sensing modalities have been used for location determination on a WLAN, such as the angle of arrival (AOA), time difference of arrival (TDOA) and radio signals from access points. The latter is one of the most commonly-used modalities, since the received signal strength (RSS) can be easily obtained from the 802.11 link layer. Hence, extra sensors are unnecessary. Currently, the fingerprinting technique is one of the most commonly used for indoor location [3,4] due to the fact that it uses RSS from each access point to estimate the device location. Localization based on fingerprinting is usually carried out in two phases. In the first phase, normally termed the offline phase, a database of the RSS from different access points at each reference location for the target environment is built. During the second phase (online phase), by means of a sample of RSS collected in a particular position and an estimation system that uses database information, the location is determined. Most research differs in the latter phase. Different algorithms and techniques, such as support vector machine [5], the ray tracing model [6], probabilistic methods [7], data mining techniques [8] or those based on the Kalman filter [9], have been developed to match the online RSS samples with the information stored in the offline database.

A major drawback of fingerprinting techniques is that the key parameter (RSS) for predicting the position of a device is not stable with time due to dense indoor multipath effects, such as reflection, diffraction and scattering. Multipath fading causes the received signal to fluctuate around a mean value at a particular location [10,11]. This effect is produced by environmental factors, such as building structures and the movement of people. Hence, a huge dataset of RSS samples is necessary to minimize this situation. Furthermore, in [12], various aspects of the RSS variance problem are analyzed when using smartphones for localization, such as device type, device placement, user direction and environmental changes over time. One of the most recent works can be found in [13] where the authors proposed a sensor fusion framework for combining WiFi, pedestrian dead reckoning and landmarks. The framework can provide an average localization accuracy of 1 m; however, in order to reach this accuracy, it has to use external references, such as landmarks, whose positions are known, to restart the algorithm when the pedestrian reaches these landmarks.

Another fundamental problem for indoor location fingerprinting is when heterogeneous wireless clients are used to measure RSS. This has a high impact on the positioning accuracy, because each client measures RSS differently. The researchers in [14] show that RSS ratios between pairs of base stations are more stable among IEEE 802.11 clients than the absolute signal strength. Hence, the memory consumption is larger, and the processing time is longer because the computations are done for pairs of base stations. Furthermore, various authors [15,16] have indicated that RSS is significantly influenced by different device orientations, and therefore, they recommend building the offline database with samples collected in different orientations in each location. At present, this is not a problem, because the new smartphones and tablets tend to have a built-in compass. Furthermore, radio mismatch problems due to the different user orientations have been studied in [17], where the authors proposed an approach that involves converting the temporal-spatial radio signal strength into a reference function, equalizing the histogram. By using equalized signals, the proposed algorithm improves the robustness of location estimation, even in the presence of mismatch orientation.

On the other hand, because lightweight mobile devices depend on battery power, the computational complexity of positioning algorithms is an essential consideration to reduce power consumption. Great progress has been made in the field of WLAN indoor localization, but most research carries out a lot of floating-point operations to predict the location, so that many computations are made; therefore, this may can cause complete battery drain within a few hours. The amount of operations depends on the number of reference locations and the number of access points located in the environment. Although several authors use some techniques to reduce floating-point operations, such as clustering [7] or localization based on two steps, where a fine localization is employed in a reduced search space determined in the first step [18,19], the number of multiplications still remains high. In our research, we proposed a localization system based on multiple weighted decision trees to predict the device location, since it has high accuracy and low computational complexity. A decision tree is a sequence of branching operations based on comparisons of RSS and orientation information. Depending on the training dataset size and the number of features, the depth of the tree can be high, and hence, the number of conditions to be evaluated could influence energy savings. Nevertheless, its computational complexity is considerably lower than the number of floating-point multiplication CPU cycles, where experimental results indicate that decimal64 multiplication with binary integer decimal (BID) encoding takes an average of 117 cycles using Intel's BID library [20]. Since no floating-point multiplication takes place to predict the location using decision trees, the computational complexity of our system is *O*(1). The latter is an extremely important characteristic if the localization system is designed for lightweight mobile devices, where both processor power and energy availability are limited.

There is no doubt that significant progress has been made in the field of indoor localization. However, an improvement to the actual methodologies is needed, giving a lower power consumption and better success ratio; this could be obtained using multiple weighted decision trees. To the best of our knowledge, our research is the first to combine RSS and device orientation with multiple weighted decision trees used for WLAN indoor localization. The main novelty of this work comes from the fact that the positioning systems based on weighted decision trees have a lower computational complexity than other research, and furthermore, their accuracy is better.

The paper is organized as follows: Section 2 explains how the model based on multiple weighted decision trees is built. Next, in Section 3, we describe our localization system, and we also analyze how it has a low computational complexity. In Section 4, we evaluate the impact of specific parameters on the performance and cost of our indoor location estimation system, and we compare the accuracy and the CPU time with other research. Finally, Section 5 contains the conclusion of this article, and future works are presented.

## 2. Multiple Weighted Decision Trees

Indoor positioning has been a very active research problem, and various data mining techniques have been used to discover useful and valid knowledge from raw data [7,8]. To solve this problem, we propose a general approach based on multiple weighted decision trees.

Decision trees build classification models in the form of a tree structure, and they can handle both categorical and numerical data. A decision tree has internal nodes and leaf nodes. An internal node includes a condition or function of any feature of the dataset, which breaks down the dataset into several subsets, corresponding to two or more branches. Each leaf is assigned to one class representing the classification decision. For instance, in the location problem, RSS and orientation are used in the internal node conditions, and the locations or reference points are used in the leaf nodes. Data collected are classified by navigating from the root of the tree down to a leaf, according to the outcome of the condition or function along the path [21].

Decision trees are generated automatically by algorithms from a given dataset. Typically, the goal is to find the optimal decision tree by minimizing the generalization error. This task is NP-hard [21], and consequently, heuristics methods are required for solving the problem, such as ID3 (Iterative Dichotomizer) [22], C4.5 [23], CART (classification and regression tree) [24], *etc*.

On the other hand, ensemble models are methods that leverage the power of multiple models to achieve better prediction accuracy than any of the individual models could do on their own. Ensemble methods generate multiple base models, and the final prediction is the combination, in some appropriate manner, from the prediction of each base model. For instance, the output of each base model is weighted. The success of the ensemble model is based on the ability to generate a set of base models that make errors that are as uncorrelated as possible. Some of the best techniques are boosting [25] and bagging [26].

In our system, we use a weak classifier based on the C4.5 algorithm to generate a decision tree as a base model. Then, the adaptive boosting (AdaBoost) algorithm [27] is used to build an ensemble model based on previous base models, that is a model formed by multiple weighted decision trees is used to estimate the location. Experimental results demonstrated that this combination of machine learning techniques is the best for our test environment. Next, we will explain how the weak classifier and the ensemble model are generated.

### 2.1. Building a Weak Classifier Based on the C4.5 Algorithm

The C4.5 algorithm takes as input a training set with instances (*x*_1_, *y*_1_), …, (*x_n_*, *y_n_*), where each *x_i_* belongs to attributes space *X* (RSS and orientation) and each label *y_i_* is in some label set *Y* (reference locations). The algorithm grows a tree using the divide-and-conquer algorithm, which is implemented recursively with the following sequence:

1. Check if the algorithm satisfies the termination criteria, that is when all instances covered by a specific branch are pure (same label value) or the number of instances falls below a certain threshold.
2. Calculate the information-theoretic criteria for all attributes. This criterion is based on the information gain, which is computed for a feature *A* as follows:
  (1) H(Y)−H(Y/A)
  where *H*(*Y*) is the entropy and *H*(*Y*/*A*) is the conditional entropy.
3. Choose the best feature according to:
  (2) $$\underset{\text{attributes}}{\max}\left[H\left(Y\right)-H\left(Y/A\right)\right]$$
4. Create a decision node with a univariate condition based on the attribute chosen in Step 3.
5. Split the dataset based on the newly-created decision node.
6. For all sub-datasets in the previous step, call the C4.5 algorithm to get a sub-tree (recursive call).
7. Attach the tree obtained in the previous step to the decision node created in Step 4.

### 2.2. Building the Model of Multiple Weighted Decision Trees

AdaBoost is the most popular boosting algorithm used for classification. It is an adaptive and iterative algorithm that combines base models of the same type, such as decision trees, in such a way that each new base model is influenced by the performance of those base models built in previous iterations. One of the main ideas of the algorithm is to maintain a weight with each instance of the training set. Initially, all weights are set equally, and the distribution of weights is denoted as *D_t_* with *t* = 1. Then, AdaBoost builds a weak classifier, *h_t_*, based on the C4.5 algorithm. Next, it uses the training set to test *h_t_*, and the weights of incorrectly classified instances are increased, so that the weak classifier is forced to focus on the hard instances in the training set. Thus, an updated weight distribution *D_t_*_+1_ is obtained. From the training set and *D_t_*_+1_, AdaBoost generates another weak classifier, *h_t_*_+1_. Such a process is repeated for *T* rounds. The final prediction is derived by weighted majority voting of the *T* weak classifiers (*h_1_*, *h*_2_, …, *h_T_*), where the weight of each classifier *α_t_* is determined during the training process by the incorrectly classified instances *ϵ_t_*. The algorithm is implemented as follows:

1. Assign equal distribution of the weights to all instances in training dataset (*x_i_*, *y_i_*) with *i* = 1,…, *n*, that is *D_t_*(*i*) = 1/*n* with *t* = 1.
2. For *t* = 1,…, *T*, do the following:
  (a) From the training dataset and *D_t_*, a weak classifier (*h_t_*: X → Y) is generated by calling the C4.5 algorithm.
  (b) Use the training dataset to test *h_t_* and measure the error as the incorrectly classified instances, that is the error is calculated as follows:
    (3) $$\epsilon_{t}=P_{i\to D_{t}}\left[h_{t}\left(x_{i}\right)\neq y_{i}\right]=\sum_{i:h_{t}\left(x_{i}\right)\neq y_{i}}D_{t}\left(i\right)$$
  (c) Calculate the weight of the base model *h_t_* as follows:
    (4) $$\alpha_{t}=\frac{1}{2}\ln\left(\frac{1-\epsilon_{t}}{\epsilon_{t}}\right)$$
  (d) Update the distribution of the instance weights as follows:
    (5) $$D_{t+1}\left(i\right)=\frac{D_{t}\left(i\right)}{Z_{t}}\times \{\begin{array}{ll} e^{-\alpha_{t}} & if\;h_{t}\left(x_{i}\right)=y_{i}\\ e^{\alpha_{t}} & if\;h_{t}\left(x_{i}\right)\neq y_{i} \end{array}$$
    where *Z_t_* is a normalization factor, which enables *D_t_*_+1_ to be a distribution, that is,
    (6) $$\sum_{i}p_{i}=1$$

The error of the final prediction is expressed as:

(7) $$\prod_{t}2\sqrt{\epsilon_{t}\left(1-\epsilon_{t}\right)}\leq e^{-2\sum_{t}{\left(0.5-\epsilon_{t}\right)}^{2}}$$

Hence, if each weak classifier is slightly better than random (*ϵ_t_* < 0.5), then the ensemble model error drops exponentially quickly.

## 3. Localization System Description

In this section, we describe our positioning system based on multiple weighted decision trees, and it is divided into two phases. The first phase is the training phase (offline phase), where RSS readings from WiFi access points at reference locations and orientation information from a compass are collected to build a database, called the radio map. From this database, the ensemble model is built. The second is the test phase (online phase) where a mobile device requests positioning services by using the online RSS and orientation information. Afterwards, we analyze how the proposed system has a low computational complexity.

### 3.1. Training Phase

**Figure 1:**
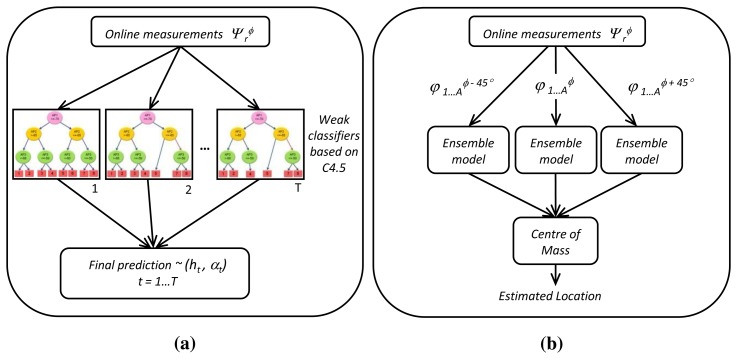
**(a)** Multiple weighted decision trees (ensemble model); **(b)** proposed system in the test phase.

The main aim of this phase is to build a model of multiple weighted decision trees starting from the dataset formed with RSS and orientation information. At each reference location, we collect the signal strength from access points in different orientations. RSS data are denoted by $\varphi_{i,j}^{\theta }\left[\tau \right]$ and indicate the τ-th RSS value measured from *i*-th access point at the *j*-th reference location with orientation *θ*, where *θ* is 0°≤ *θ* < 360°. The radio map can be represented by *Ψ* as follows:

(8) $$\psi^{\theta }=\left(\begin{array}{cccc} \varphi_{1,1}^{\theta }\left[\tau \right] & \varphi_{1,2}^{\theta }\left[\tau \right] & \cdots & \varphi_{1,R}^{\theta }\left[\tau \right]\\ \varphi_{2,1}^{\theta }\left[\tau \right] & \varphi_{2,2}^{\theta }\left[\tau \right] & \cdots & \varphi_{2,R}^{\theta }\left[\tau \right]\\ \vdots & \vdots & \ddots & \vdots \\ \varphi_{A,1}^{\theta }\left[\tau \right] & \varphi_{A,2}^{\theta }\left[\tau \right] & \cdots & \varphi_{A,R}^{\theta }\left[\tau \right] \end{array}\right)$$

where *A* is the number of access points, *R* is the number of reference locations, *τ* = 1, …, *N* is the index of temporal RSS samples and *N* is the number of RSS samples gathered at each reference location. The same values of orientation *θ* must be taken at each reference location, for instance, 0°, 45°, 90°, and so on. The ensemble model is formed by *T* different decision trees built with the C4.5 algorithm (Figure 1a).

The size of the dataset influences the performance of the localization system and the time taken to build and evaluate the ensemble model of decision trees. A detailed analysis of the training size and the optimal number of decision trees is explained in Section 4.

### 3.2. Test Phase

In this phase, measurements from access points and a digital compass are taken as input, and the current location is predicted. Using similar notations, the online measurements can be represented as follows:

(9) $$\psi_{r}^{\theta }=\left(\begin{array}{c} \varphi_{1,r}^{\theta }\left[q\right]\\ \varphi_{2,r}^{\theta }\left[q\right]\\ \vdots \\ \varphi_{A,r}^{\theta }\left[q\right] \end{array}\right)$$

where the location *r* is unknown, *θ* is the orientation angle of sampling and *q* is the number of consecutive RSS samples collected in *r*. Since the radio map was built with different values of orientation spacing 45° and the online orientation gathered by the digital compass can vary between 0°and 360°, we must adapt the real-time orientation value to one of these values. On the other hand, a digital compass can lose its calibration when it is close to electromagnetic objects and electronic devices. Hence, the nearest orientation to a multiple of 45° might not always be the best solution in order to adapt the real orientation. We have done three different experiments to evaluate the best performance of the positioning system by varying the *θ* feature.

1. Nearest orientation: The estimated location is predicted by rounding the value taken by a digital compass to the nearest multiple value of 45°. That is, if the digital compass returns a value equal to 78°, it will be rounded to 90°.
2. Center of mass: The estimated location is calculated as the center of mass of a triangle whose vertices are the coordinates of the predicted positions. These three coordinates are the result of evaluating the ensemble model of decision trees with three different orientations. The values of these orientations are the nearest orientation, the nearest orientation plus 45° and the nearest orientation minus 45°. The center of mass is computed as follows:
  (10) $$\left(x_{c},y_{c}\right)=\frac{1}{3}\sum_{i=1}^{3}\left(x_{i},y_{i}\right)$$
  where (*x_i_*,*y_i_*) is the coordinate of the predicted location of each ensemble model.
3. Nearest vertices: The estimated location is calculated as the arithmetic mean of the two nearest vertices of a triangle whose vertices are the same as the previous experiment.

**Figure 2:**
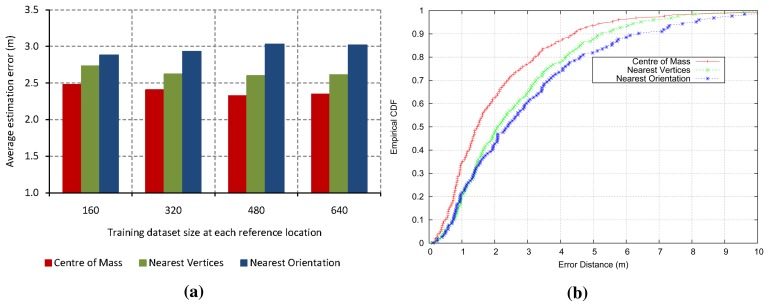
Evaluation of center of mass, nearest vertices and nearest orientation methods: (**a**) location estimation error; (**b**) CDF of performance.

Figure 2a shows the average location estimation error for every experiment with regard to the training size at each reference point, which is computed as the arithmetic mean of several experiments. Eight different orientations (spacing 45°) were taken in 166 reference locations. Therefore, the total training dataset size is given by the multiplication of the samples collected at each orientation and location, the amount of different orientations and the number of reference locations. Figure 2b shows the cumulative distribution function (CDF) for each experiment. The error is the expected distance from the device's actual location to the predicted location. As we can see, the center of mass method achieves better accuracy than others, because it minimizes the high variability reported by the digital compass and, furthermore, provides a more robust and accurate approach. Hence, we proposed a test phase based on the centroid, that is the test phase of our localization system will evaluate the ensemble model of decision trees three times. After that, the center of mass of three predicted locations is calculated to obtain the coordinates of the final estimated position. Figure 1b shows the proposed system in the test phase.

### 3.3. Analysis of the Computational Complexity

In order to predict a location, a computation based on the online measurements, as well as the model execution is carried out. If the calculations take place on a lightweight mobile device, the localization system has to be efficient with regard to CPU and memory usage. High computational algorithms can cause complete battery drain within a few hours. Most well-known research has a computational complexity similar to *O*(|AP| × |RL|), where |AP| is the number of access points and |RL| is the number of reference locations. This is because the number of multiplications is correlated to the number of access points at each reference point. Some algorithms use clustering techniques to reduce the number of reference points to explore. Despite this improvement, the number of multiplications still remains high.

In contrast, the computational complexity of our location system is *O*(1), because no floating-point operation takes place on the ensemble model of decision trees. A sequence of branching operations based on comparisons of received signal strength and orientation information are only carried out when walking along the decision tree. Furthermore, a constant number of floating-point operations will only take place when the center of mass is calculated. Specifically, six floating-point operations are carried out. Hence, the computational cost of the localization system presented in this paper is very low. Experiments demonstrating the energy savings of our localization system are detailed in the next section.

## 4. Experimental Results and Discussion

In this section, we describe the test environment, and we evaluate the impact of specific parameters on the performance of our indoor location estimation system. Afterwards, we compare the accuracy and the computational cost of our system with Horus [7], Radar [15], Rice [28] and Compass [29] approaches. Although all experiments were carried out on an Intel Core 2 Duo 2.66 GHz/4 GB RAM non-dedicated Windows machine, the results can be easily extrapolated to a lightweight device, such as a smartphone or tablet. The error distance between the estimated location and the real position is calculated by the Euclidean distance between these points, and the arithmetic mean was computed from the results of the experiments.

### 4.1. Test Environment

Indoor location estimation based on multiple weighted decision trees was evaluated on the second floor of an office building at Mannheim University, Germany. The traces of signal strength of 802.11 access points and orientation information were downloaded from the Crawdad public database [30].

**Figure 3:**
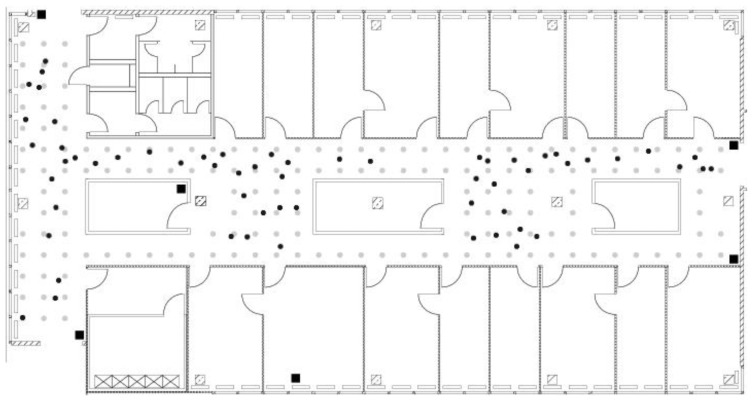
Layout of the testbed floor at University of Mannheim. The gray dots represent the offline reference locations; the black dots represent the selected online locations; and the squares show the location of access points.

The floor area is nearly 15 m wide and 36 m long, covering an area of approximately 312 square meters. The floor plan of the testing area is shown in Figure 3. The large hallway on the left-hand side of the map is connected by two narrow hallways that are separated by several rooms. The test environment is equipped with five Linksys WRT54GS and four Lancom L-54g access points. All access points support 802.11b and 802.11g. One Lancom and all Linksys access points are located on the same floor as the testing area, whereas three Lancom access points are located in other places inside the building. The exact position of the access points located inside the testing area is marked by squares on the floor plan.

The grid of reference points applied to the operation area includes 166 reference locations (gray dots in Figure 3) with a spacing of 1 m. They are equally distributed in the radio map. During the offline phase, the signal strength was measured at each reference location in eight different orientations (spacing of 45°). One hundred ten signal strengths and orientation measurements were collected at each reference location. Consecutive measurements were taken every 250 ms. This leads to 146,080 measurements for the offline phase.

**Figure 4:**
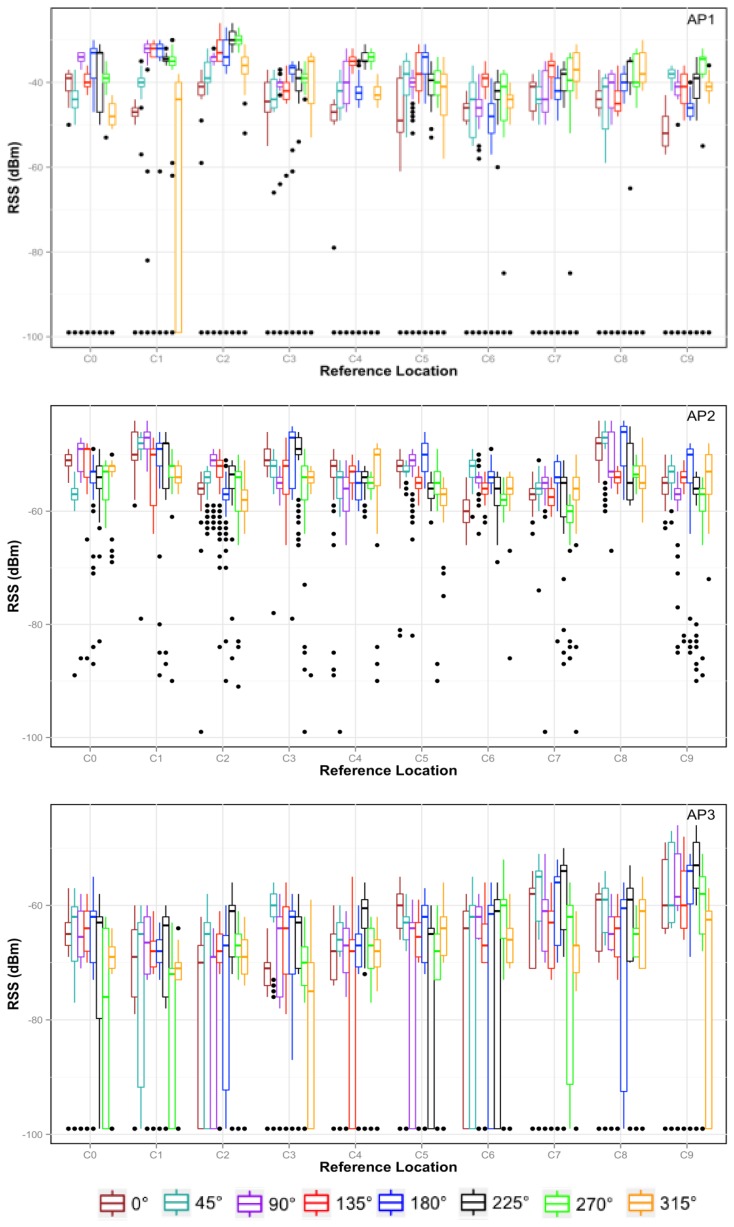
Data distribution for ten locations and the AP1, AP2 and AP3 access points.

The data distribution for the first ten reference locations and for three access points is shown in Figure 4 in the form of boxplots. As can be observed, the RSS varies for each access point when changing the location and even for the same location when changing the orientation. For instance, in location C0 the highest RSS corresponds to AP1 when the device is facing south (180°), but facing northwest (315°), the RSS can be about 10 dB lower. The RSS variance with orientation does not have a similar behavior in all locations. For that reason, our approach uses a sensor fusion combining RSS and orientation, because this fusion yields an even more accurate system.

On the other hand, 60 locations were randomly selected (black dots in Figure 3) for the online phase. One hundred ten consecutive RSS and orientation measurements were also collected at each test location. This leads to 6600 measurements for the online phase.

In both cases, a Lucent Orinoco Silver PCMCIA network card supporting 802.11b was used to gather the received signal strength. The orientation was obtained with the Silicon Laboratories C8051F350 Digital Compass Reference Design Board, which was calibrated in the middle of the operation area.

### 4.2. Trace Preprocessing of the Training Dataset

All models of multiple weighted decision trees of our experiments have been built using the API Weka software [25]. Weka is an open source collection of machine learning algorithms for data mining tasks, more specifically data preprocessing, clustering, classification, regression, visualization and feature selection. The classification base model tree was created by the C4.5 algorithm [23] (implemented in Weka by the classifier *weka.classifiers.trees.J48*). The boosting method used was the meta learning AdaBoostM1 algorithm, an extension of the AdaBoost to the multiclass case, with more than two possible classes, designed specifically for classification (implemented in Weka by the classifier *weka.classifiers.meta.AdaBoostM1*). Weka uses an ARFF (Attribute-Relation File Format) file to describe a list of instances sharing a set of attributes. Hence, in order to use the dataset from the University of Mannheim with the API Weka, we had to parse it and generate an ARFF file, for both the offline phase and the online phase.

On the other hand, as we have said above, the RSS was measured at each reference location in eight different orientations with a digital compass. Nevertheless, if we analyze the trace entries of the Mannheim dataset, we can observe that the RSS were not collected exactly at every 45°. For example, in a specific reference location with the orientation 90°, the value measured was 90.6°, and in another reference location, it was 90.1°. These minimal differences are found in all reference locations and for every orientation of the dataset. In this situation, the C4.5 algorithm considers each value of the orientation as a possible state, and therefore, multiple leaves of the decision tree are created. This represents a decision problem, since a classification is generated for every value of orientation, and therefore, a problem of overfitting can appear in the training phase. From the previous example, a possible location is generated for the orientation 90.6° and another location for 90.1°, and in those conditions, the reference location could be exclusively determined from the orientation, independently of the RSS, if orientations were unique for each reference location. However, both values represent the same value of the orientation, 90°. Furthermore, the orientation information in online traces will probably not match with it, so that the location estimation may be highly erroneous. In fact, in the online phase, the orientation is rounded to the nearest multiple value of 45°; see Section 3.2. Hence, we set the value of the orientations exactly to 0°, 45°, 90°, 135°, 180°, 225°, 270° and 315°, where 0° means that the device is facing north.

It is also possible that some access points may not be detected at certain reference locations due to the characteristics of signal propagation. Because decision tree algorithms need a value for each access point, an RSS default value was assigned to every missing access point. For those cases, the RSS value was set to −99 dBm, which is below the WiFi receiver sensitivity (−94 dBm in our network card at best), because it is below the minimal RSS value measureable by the device, such as that used in [31].

Lastly, attribute selection techniques were implemented in Weka, such as principal component analysis and correlation-based feature selection (known as *CfsSubsetEval* in Weka) to reduce the dimensionality of the datasets. The former technique returned that all access points were the most representative features, and the latter reported that the access point AP8 was not selected as a relevant feature. Nevertheless, experimental results demonstrated that better results were achieved with all of the features, that is all access points and orientation information; see Table 1. From the results, and as was expected, the orientation attribute has a significant contribution to the accuracy. Hence, all access points and orientation features were used in all of the experiments.

### 4.3. Analysis of the Training Size

The size of the training dataset is an important parameter for the performance and the building time of each decision tree. A large-sized training dataset can provide better accuracy to predict the correct location, but too much data can increase the elapsed time to build the model of multiple weighted decision trees considerably. However, we must take into account that the model is generated only once in the offline phase, and it can be built using a dedicated supercomputer. On the other hand, an ensemble model of decision trees can be built in a short time for a small-sized dataset, but the accuracy of the system may not be good.

In order to evaluate our localization system with the training size, we generated the ensemble models by varying this value between 40 and 640 instances or traces at each reference location (between 5 and 80 traces at each orientation and reference location), with *T* equal to 10. This number of instances is randomly chosen from the 110 measurements collected in the offline phase. In Table 2, the relationship between this value and the number of total instances used for training is shown. For the test phase, we randomly chose ten consecutive traces with the same orientation in the online phase. The arithmetic mean of RSS values of these traces for each access point was calculated and used as input for the ensemble model. For the validity of experimental results, we performed this experiment 6000 times for each training size.

**Figure 5:**
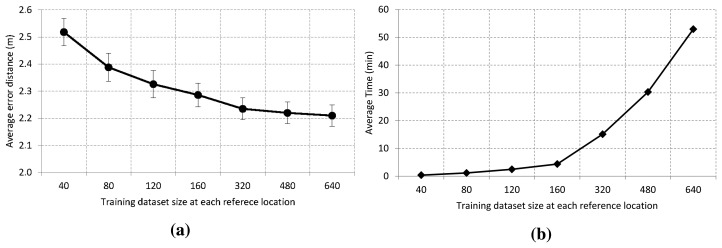
Effect of the training size at each reference location on the: (**a**) performance of the proposed system; (**b**) elapsed time to build the model of multiple weighted decision trees.

Figure 5a shows the effect of the training size at each reference location on error distance with a 95% confidence interval. As we can see, the ensemble model accuracy is enhanced as we increase the training size until it reaches a stable value. The average error is about 2.5 m when we use an ensemble model of decision trees based on 40 traces at each reference location. Furthermore, the accuracy improves around 10 cm if the training size is increased from 40 to 80. After that, there is a slight improvement for sizes of 120, 160 and 320 traces. Then, the accuracy remains approximately stable at about 2.2 m when the size is increased to 480 and 640 traces. The low confidence interval, ±0.045, is related to the reliability of the estimation procedure.

Figure 5b shows the elapsed time to build the model of multiple weighted decision trees when the training size varies between 40 and 640 traces at each reference location. As we can see, the elapsed time grows very quickly in a non-linear way. It is less than 5 min for a size equal to 160 samples, and it is about 53 min for a size equal to 640. Although this time difference may seem important and meaningful, we must take into account that the model is generated only once.

On the other hand, Table 2 shows the arithmetic mean of the decision tree depth built with the C4.5 algorithm. As can be observed, the number of leaves grows linearly with the training dataset size. Nevertheless, the average tree depth is increased by three comparisons from the smallest to the largest dataset. In the worst case, the maximum average number of comparisons is only 16, and hence, it has a very low computational complexity. Based on these results, we use 640 samples as a training size at each reference location for most of our experiments, that is a total dataset size of 106,240 traces.

### 4.4. Analysis of the Test Size

As is well known in indoor environments, the RSS at a given location varies with time, since the signal propagation depends on the building distribution and the materials that these are made of, furniture, as well as people roaming around. These factors generate a multipath effect caused by reflection, diffraction and signal scattering. Therefore, due to the RSS variance problem, RSS samples obtained during the online phase may deviate from those stored in the radio map database. As a result, this deviation may lead to an estimation error of the position.

In order to minimize the variation of RSS with time, the arithmetic mean from a set of consecutive RSS collected with the same orientation is used as input for the ensemble model. Hence, the variation of RSS is smoothed out. We termed the test size to the number of consecutive samples collected at a test location and averaged them to generate the input sample.

**Figure 6:**
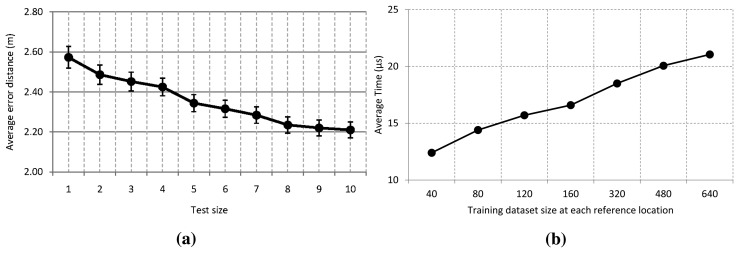
(**a**) Effect of the test size on the performance of the system; (**b**) effect of the training size on the elapsed time to estimate a location.

In order to evaluate our localization system with the test size, we have varied this value between 1 and 10. We chose a training size of 640 to build the model with *T* equal to 10. For the validity of the experimental results, we performed this experiment 6000 times for each test size. The effect of this parameter on the performance of the ensemble model with a 95% confidence interval is shown in Figure 6a. The graph shows that an average error (arithmetic mean) close to 2.6 m is obtained even if just one trace is used. From three to five samples, the error distance decreases at least 20 cm. As can be seen in the graph, any additional trace improves the accuracy of the system, although the improvement tends to stabilize around 2.20 m.

With regard to the elapsed time to get a prediction, we did not evaluate the test size, because this only implies calculating the arithmetic mean of a set of consecutive samples. For that reason and because it has a better accuracy, we use 10 consecutive measurements as a test size for most of our experiments, and the arithmetic mean of these is used as input. Figure 6b shows the elapsed time to get a prediction in our location system with a test size of 10, where we have varied the training size at each reference location between 40 and 640 traces. As expected, the elapsed time to get a prediction increases with the training sample size. Nevertheless, this not meaningful, because it only varies nine microseconds from the smaller to larger size of the dataset. This is because the depth of the tree grows slightly with the training size, and some more comparisons have to be made at each decision tree.

### 4.5. Analysis of the Optimal Number of Decision Trees

**Figure 7:**
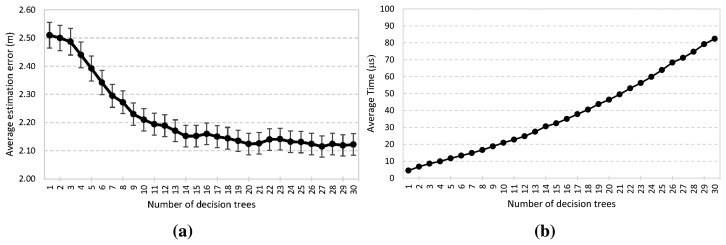
Effect of the multiple decision trees: (**a**) average estimation error; (**b**) elapsed time.

As mentioned in Section 2.2, the ensemble model is built with *T* weighted decision trees. When more decision trees are used, the proposed system can be more robust. Figure 7a,b reports the accuracy performance and the elapsed time to determine a location when the number of decision trees changes. It is demonstrated that arithmetic mean error decreases as *T* increases (Expression 7). When the number of decision trees is increased from 1 to 10, a significant performance improvement can be observed. However, the improvement fades away gradually when the number of decision trees used is larger than 11. Furthermore, in the case of a larger *T*, the proposed system generates more stable average errors for localization, reaching the minimum value several times, 2.1 m with *T* = 20, 21, 27, 29 and 30. Furthermore, a 95% confidence interval is shown. The confidence interval decreases as *T* increases, with the minimum value being about ±0.038. On the other hand, from Figure 7b, the results demonstrate a trade-off between the online computation and accuracy. The average time to determine a location increases linearly as *T* does. Nevertheless, it only involves an increase in the number of comparisons, and therefore, the computational complexity remains *O*(1) regardless of *T*. In the worst case scenario, it is an order of magnitude of microseconds and is faster than other positioning systems, as will be shown in the next section.

### 4.6. Accuracy and Computational Cost

In this section, we analyze the performance of our localization system with the performance of the Horus, Radar, Rice and Compass positioning systems. The Loceva tool [32] implements these systems, and we have used it to evaluate the different approaches. We set the training size to 106,240 traces, that is, 640 samples at each reference location, the model was built with *T* equal to 10 and the test size was fixed to 10 in all positioning systems to carry out the evaluation in equal conditions. Furthermore, we set some parameters of the other positioning systems as follows, because these parameters achieved the best performance for the test environment. For the Radar system, we used the NearestNeighbors algorithm with k equal to four. For the Horus system, the value of the ClusterKey parameter was set to one. For the Compass approach, we set the *k* parameter to five and the *α* parameter to 180.

**Figure 8:**
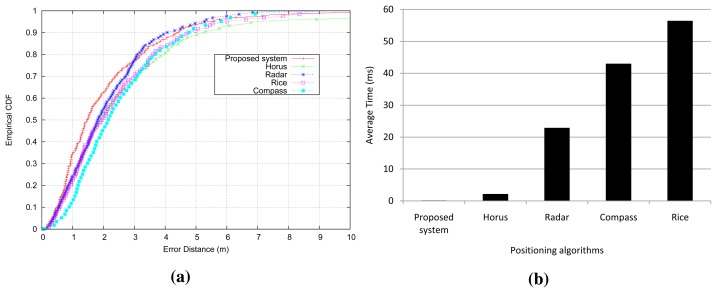
Evaluation of different positioning algorithms: (**a**) CDF of performance; (**b**) elapsed time to estimate a location.

Figure 8a shows the performance of the analyzed systems. As we can see, all of the approaches have a similar accuracy. Nevertheless, our localization system is slightly more accurate than the rest of the positioning systems. Specifically, the success probability is more accurate from one meter to three meters, and the accuracy of the proposed system is comparable to other techniques at the 80th percentile and above. On average, our location system achieves an error distance of 2.21 m in comparison to the 2.52 m of Compass, 2.50 m of Horus, 2.42 m of Rice and 2.25 m of Radar.

With regard to the elapsed time to get a prediction in each positioning system, our approach is faster than other algorithms because our approach uses decision trees to predict the location, which only requires comparison operations when walking along the tree. However, the rest of the positioning systems require the multiplication of conditional probabilities in proportion to the number of access points at each reference location. Only the Horus system reduces the number of multiplications, because it uses a clustering technique to group reference locations based on the access points covering them. The results are summarized in Figure 8b. The time elapsed by the other algorithms is measured in milliseconds, and the Horus system is the fastest of them, with an average time of 2.2 ms. Nevertheless, our localization system takes an average time of about 21 µs at worst from Figure 6b. Hence, the proposed system is about 100-times faster than Horus, and it is more than 1000-times faster than the other positioning algorithms.

On the other hand, a comparison among different classification methods was carried out in order to demonstrate the effectiveness of boosting. The methods compared were the following: *k*-Nearest Neighbor with *k* = 1, the C4.5 algorithm, bagging using C4.5 [33] and boosting using C4.5 (used in our approach). C4.5 trees were pruned to avoid overfitting, and better results were reached with ConfidenceFactor *C* = 0.25. A value of *T* = 10 was used in bagging and boosting multiclassifiers, and for the validity of experimental results, the experiments were carried out using 10-fold cross-validation.

Table 3 shows the achieved results in terms of correctly classified instance percentage for each training size. It can be noticed that 1-NN outperforms the single classifier C4.5, but boosting outperforms all of the methods overall (71.99%).

## 5. Conclusions

In this paper, we have proposed a low complexity positioning system based on multiple weighted decision trees. Data fusion based on RSS from access points and orientation information from a digital compass is used to build the ensemble model. We have carried out the experiments with a public dataset from the University of Mannheim. Our localization system is considerably faster than other well-known positioning systems, such as Horus, Radar, Compass and Rice. In the worst case, it is about 100-times faster than Horus, but it is more than 1000-times faster than other positioning systems. With regard to accuracy, our proposed approach achieves an average error distance of 2.1 m with a number of weighted decision trees equal to 20, which is better than the other positioning systems. Furthermore, to minimize the high variability reported by the digital compass when it is close to electromagnetic objects and electronic devices, we used the center of mass to get the final prediction, which is computed from the prediction of three ensemble models. This provides a more robust and accurate approach.

On the other hand, the accuracy of the model of multiple weighted decision trees improves with the training size, and its effect on the elapsed time to get a prediction is not meaningful, because the average tree depth goes only from 13 to 16 comparisons in the worst case. Furthermore, we analyzed and determined the optimal number of weighted decision trees and concluded that an average of consecutive traces used as input in the ensemble model improves the accuracy of the positioning system, because it minimizes the RSS variance.

In summary, the main contributions of this work are:

- We proposed an indoor localization system based on multiple weighted decision trees (boosting), which is effective and computationally light (low complexity), so that the system can be run on a resource-limited mobile device without compromising the battery life.
- We used a sensor fusion system for combining RSS and orientation for indoor localization, and we demonstrated an average localization accuracy of 2.1 m.
- A center of mass is used to improve the accuracy and to minimize the RSS variance.

In environments with a high density of access points the decision trees size grows in such way that the elapsed time to estimate a location is penalized. Hence, in the future, we will consider incorporating clustering and feature selection techniques when complex environments are handled. In this way, decision trees with few leaves will be defined in each cluster (low complexity), and redundant signals from some access points should be removed.

On the other hand, since visible light communication (VLC) is experiencing growing interest due to improvements in solid state lighting and high demand for wireless communications, in our ongoing work, we are also planning to use the decision trees to predict device location in a communication network based on the recently approved standard IEEE 802.15.7. Since this kind of network does not have electromagnetic interference and the received optical power is more stable than radio signals, VLC offers new opportunities in carrying out accurate indoor positioning.

## Figures and Tables

**Table 1:** Correctly classified instances for different features selection using boosting with C4.5.

**Features**	**Accuracy Results** (%)
All APs and orientation	**71.99**
All APs and orientation except AP8	71.39
All APs and without orientation	49.16
All APs except AP8 and without orientation	48.09

**Table 2:** Decision tree depth *vs.* the training dataset.

**Samples at Each Orientation**	**Samples at Each Reference Location**	**Training Dataset Size**	**Min Depth**	**Max Depth**	**Average Depth**	**Leaves**
5	40	6640	6	21	13	2171
10	80	13,280	6	24	14	3908
15	120	19,920	6	25	14	5420
20	160	26,560	7	25	15	6812
40	320	53,120	7	25	16	11,317
60	480	79,680	6	28	16	15,398
80	640	106,240	7	27	16	19,105

**Table 3:** Correctly classified instances (%) for different classification methods and training size.

**Algorithm**	**Traces at Each Reference Location**						

	**40**	**80**	**120**	**160**	**320**	**480**	**640**
1-NN	23.68	33.22	39.25	43.42	53.20	57.84	60.73
C4.5	17.06	27.43	32.99	38.01	49.37	54.71	57.29
Bagging C4.5	24.81	37.93	45.96	51.92	61.32	65.66	68.11
Boosting C4.5	26.98	41.36	49.82	55.04	65.63	69.77	**71.99**
